# Water Pollution

**Published:** 1913-01

**Authors:** 


### Water Pollution

A year ago, we discussed the Buffalo water supply. In October 1911, we discussed the Laurentian system of water ways. The first article in the present issue deals with the problem from the Canadian standpoint but in so broad a spirit that it throws light on the whole subject. While depending upon the same bacteriologic principles, the problem with regard to a well, spring, or water shed for a family or small community, presents quite different details in engineering and sanitation, economics and law, from that involving a large body of fresh water, although it must be conceded that most insignificant sources of water are tributaries to larger ones and, hence, gradually and remotely, approach the latter in this respect.

It is fortunate that the Laurentian problem is international in scope. While, at first sight, the necessity of joint action by two governments seems to be an obstacle to progress, further reflection convinces us that we are rapidly nearing the point at which the menace of the life and health of citizens of one country by the pollution of boundary waters by the citizens of another country must be considered an aggressive act, persistence in which would justify war quite as clearly as aggressions by traumatic measures or aggressions against property. We speak in no spirit of jingoism and hasten to add that the friendship and enlightenment of both countries preclude any such possibility, quite aside from the fact that the interests of each country are the same. Thus, we feel confident that the international scope of the problem will expedite its solution.

